# The effects of the administration sequence and the type of hypnotics on the development of remifentanil-induced chest wall rigidity: a randomized controlled trial

**DOI:** 10.1186/s12871-023-02154-5

**Published:** 2023-06-08

**Authors:** Yu Jin Oh, Yesull Kim, Chanhong Lee, Dong-Chan Kim, Aram Doo

**Affiliations:** 1 Department of Anesthesiology and Pain Medicine, Jeonbuk National University Medical School and Hospital, 20, Geonji-Ro, Deokjin-Gu, Jeonju, Jeollabuk-Do 54907 South Korea; 2 Research Institute of Clinical Medicine of Jeonbuk National University–Biomedical Research Institute of Jeonbuk National University Hospital, Jeonju, South Korea

**Keywords:** Complication, Elderly, General anesthesia, Hypoxemia, Opioids, Remifentanil, Respiratory failure, Rigidity

## Abstract

**Background:**

Research on remifentanil-induced chest wall rigidity is limited. Furthermore, its incidence is unknown, and the clinical factors influencing its development remain unclear. This prospective, double-blind, randomized controlled trial aimed to investigate the effects of the administration sequence of hypnotics and remifentanil as well as the type of hypnotic administered on the development of remifentanil-induced chest wall rigidity.

**Methods:**

A total of 125 older patients aged $\ge$ 65 years, who were scheduled to undergo elective surgery under general anesthesia, were enrolled in this study. Participants were randomly assigned to one of four groups; Thio-Remi, Pro-Remi, Remi-Thio, or Remi-Pro. After confirming the loss of consciousness and achieving a target effect-site concentration of 3 ng/mL remifentanil, the development of remifentanil-induced chest wall rigidity was evaluated.

**Results:**

The incidence of chest wall rigidity was significantly higher in the remifentanil-hypnotic group than in the hypnotic-remifentanil (opposite sequence) group (55.0% vs. 21.7%, *P* < 0.001). Logistic regression analysis revealed that remifentanil-hypnotic administration was a significant predictor of the development of chest wall rigidity (crude odds ratio 4.42, 95% confidence interval 1.99; 9.81, *P* < 0.001).

**Conclusions:**

Pretreatment with hypnotics potentially reduces the development of chest wall rigidity during the induction of balanced anesthesia with remifentanil in older patients.

**Trial registration:**

This article was registered at WHO International Clinical Trials Registry Platform (Trial number: KCT0006542).

## Background

Opioids have evidently been one of the major drugs for anesthetic induction and maintenance. They provide intra- and post-operative analgesia and promote hemodynamic stability during surgery and airway manipulation by blocking sympathetic stimulation [1]. Remifentanil is an ultra-short-acting synthetic opioid, which is a widely used adjuvant for balanced anesthesia. Remifentanil may improve the quality of anesthesia by reducing hypnotic dose requirements and shortening the onset of anesthesia during anesthesia induction [2]. Its pharmacokinetic characteristics and short context-sensitive half-life potentially enhance rapid recovery from anesthesia compared with other opioids, such as fentanyl [2]. These characteristics render it more favorable for fast-track anesthesia and surgery. In addition, remifentanil is metabolized by nonspecific tissue esterase; therefore, it can be safely used in patients with underlying diseases, such as hepatic or renal dysfunction [3].

Intravenous administration of a bolus of opioids during anesthesia induction frequently elicits unexpected adverse effects, including cough, muscle rigidity, laryngospasm, and resultant respiratory failure [4–6]. In particular, opioid-related muscle rigidity is most prominent in the chest and abdomen. Chest wall rigidity followed by opioid administration, also called “wooden chest syndrome” may be one of the most serious complications [7]. Opioid-induced chest wall rigidity may worsen lung compliance, thereby causing ineffective ventilation. It potentially leads to life-threatening hypoxemia, hypercarbia, and desaturation [5, 8]. Although it is clinically important for patient safety, there have been few well-designed clinical trials that characterized it. Potential risk factors includes high-dose opioids administration and extremes of age (neonate or elderly), and critical neurologic or metabolic illness [8–11].

A few studies have investigated chest wall rigidity due to remifentanil administration [6, 12, 13]. However, its incidence is unknown, and the clinical factors that affect it remain unclear. Meanwhile, older patients present with hypoxemia and desaturation during anesthesia more frequently because this population has reduced pulmonary reserve [14]. Therefore, the consequences resulting from the abrupt development of chest wall rigidity are potentially more complicated in this population.

This prospective, double-blind, randomized controlled trial aimed to investigate the development of remifentanil-induced chest wall rigidity in older patients (≥ 65 years) undergoing general anesthesia. We hypothesized that the administration sequence of hypnotics and remifentanil (order of administration: hypnotic-remifentanil versus remifentanil-hypnotic) during anesthesia induction potentially affects the development of remifentanil-induced chest wall rigidity. We also compared the effects of different types of hypnotics, including thiopental sodium and propofol, which are the most commonly administered intravenous anesthetics. This study also aimed to determine the predictive factors for chest wall rigidity in older patients.

## Methods

This study was approved by the Institutional Review Board of the authors’ hospital and registered with the WHO International Clinical Trials Registry Platform (Trial number: KCT0006542, Date of registration: 03/09/2021). The protocol is available by reasonable request from the corresponding author. This manuscript adheres to the applicable CONSORT guidelines. Participants were recruited from a tertiary care teaching hospital. After obtaining written informed consent, 125 older patients aged $\ge$ 65 years with American Society of Anesthesiologists physical status 1 or 2, who were scheduled to undergo elective surgery under general anesthesia, were enrolled in this study. Participants who had a history of allergy to propofol or thiopental; severe pulmonary disease including asthma, chronic obstructive pulmonary disease or interstitial lung disease; severe hepatorenal impairment; or had taken opioid analgesics within 3 months prior to surgery were excluded from the study.

All participants enrolled in the study underwent body composition measurements using a body composition analyzer (Inbody S10® Biospace, Seoul, Korea) the day before surgery. Body composition measures, such as body fat mass (kg), percent body fat (%), skeletal muscle mass (kg), skeletal muscle mass index (kg/m^2^), and the amount of total body water (L), were evaluated.

### Group allocation

Participant group allocation was based on the administration sequence of hypnotics and remifentanil (hypnotic-remifentanil and remifentanil-hypnotic groups), and further subdivision was based on the type of hypnotic administered, that is, thiopental or propofol. Consequently, participants were randomly assigned to one of four groups in a 1:1:1:1 ratio using computer-generated random numbers. In the Thio-Remi group, thiopental was administered first until loss of consciousness (LOC), followed by remifentanil infusion via target-controlled infusion (TCI) at an effect-site concentration (Ce) of 3 ng/mL. In the Pro-Remi group, propofol was administered first until LOC, followed by remifentanil at a Ce of 3 ng/mL. In the Remi-Thio group, remifentanil was first infused at a Ce of 3 ng/mL using a TCI pump, followed by thiopental until LOC. In the Remi-Pro group, remifentanil and propofol were administered in that order and in the same manner.

### Drug preparation, blinding, and randomization

Thiopental sodium (500 mg), diluted with 20 ml of normal saline at a concentration of 25 mg/mL, was prepared in a 20-ml syringe. Twenty milliliters of propofol (1%) was prepared in a 20-mL syringe. Remifentanil (1 mg) was diluted with normal saline to a concentration of 50 ug/mL (final volume, 20 ml). To maintain blinding of the outcome assessor (an experienced anesthesiologist, Y. J. Oh), syringe contents (thiopental, propofol and remifentanil) were concealed with an opaque syringe and giving sets. All anesthetic drug infusions during the intervention were blinded to both the patient and outcome assessor, and all drugs used for the intervention were prepared by a single anesthetic nurse. Randomization was performed using computer-generated random numbers by an anesthesiologist who was not involved in the anesthetic intervention and data collection.

### Anesthetic regimen

The anesthetic regimen was standardized for all participants. None of the patients received any preanesthetic medication. Standard anesthetic monitoring, including electrocardiography, noninvasive blood pressure, pulse oximetry, capnography, and bispectral index, was implemented.

In the hypnotic-remifentanil groups, that is, the Thio-Remi and Pro-Remi groups, thiopental sodium or propofol was initially infused at a rate of 350 mL/h using an infusion pump (Agilia®, Fresenius Vial, France) until LOC was achieved (no response to verbal command “open your eyes”). Thereafter, the target Ce was set at 3 ng/mL using a TCI pump (Orchestra® Base Primea, Fresenius Vial, France), in which the Minto model was pre-programmed, in effect-site targeting mode. Remifentanil was initially infused using a TCI pump in the remifentanil-hypnotic groups (Remi-Thio and Remi-Pro groups). When a predetermined Ce of 3 ng/mL was achieved, thiopental or propofol was administered in the same manner as in the hypnotic-remifentanil groups.

The total dose of hypnotics administered until LOC and elapsed time to LOC were recorded. Moreover, vital signs, including peripheral oxygen saturation (SpO_2_), blood pressure, heart rate, and bispectral index, were monitored during the intervention.

### Evaluation of chest wall rigidity

After confirming loss of spontaneous breathing and achieving a target Ce of 3 ng/mL remifentanil, the development of remifentanil-induced chest wall rigidity was evaluated for 120 s while providing manual positive pressure ventilation without administration of a neuromuscular blocking agent. During the assessment, the fresh gas flow rate and inspired fraction of oxygen were set at 6 L/min and 0.5, respectively. An experienced anesthesiologist (Y. J. Oh) blinded to the study groups assessed the patients using a 3-point Likert scale (1 = Unlikely, 2 = Probable, and 3 = Definite) as follows: Grade 1 (Unlikely); no signs of chest wall rigidity during positive pressure ventilation; Grade 2 (Probable); increased inspiratory airway pressures required to deliver appropriate tidal volumes (6–8 mL/kg *ideal body weight*), with SpO_2_ maintained at or above 94%; and Grade 3 (Definite); decreased tidal volumes (< 6 mL/kg *ideal body weight*), despite an increased peak inspiratory airway pressure of up to 35 cmH_2_O and the development of SpO_2_ desaturation < 94%. To qualify as an event, the development of chest wall rigidity had to be evaluated for at least five consecutive ventilatory cycles. After the assessment was completed, rocuronium (1 mg/kg) was administered to facilitate endotracheal intubation. If SpO_2_ desaturation < 94% did not resolve after successful endotracheal intubation, an incremental dose of naloxone 0.1 mg was intravenously administered. Grade 2 or 3 was defined as the development of remifentanil-induced chest wall rigidity.

The primary outcome was the incidence of remifentanil-induced chest wall rigidity. The development of other adverse effects, such as desaturation and cough, during the intervention was also compared as secondary outcomes. Potential risk factors for the development of remifentanil-induced chest wall rigidity were also identified.

### Statistical analysis

The sample size was predetermined by the Chi-square Sample Size calculation using SigmaPlot 14.5 (Systat Software Inc. San Jose, USA) based on the assumption that the incidence of remifentanil-induced chest wall rigidity, which was considered the primary endpoint, would be 15% and 45% in the hypnotic-remifentanil and remifentanil-hypnotic group, respectively. A total of 103 patients were required, with a significance level of 0.05 (α = 0.05) and power of 80% (β = 0.20). To allow attrition, the total sample size was increased to 125.

All statistical analyses were performed using SigmaPlot 14.5. Continuous variables were compared using one-way analysis of variance (ANOVA) with Tukey post-hoc test after normality test using Shapiro–Wilk test. Categorial variables were analyzed using chi-square test. Vital signs, including mean arterial pressures and heart rate, were analyzed using two-way repeated measures ANOVA. The Holm-Sidak method and Bonferroni correction were used for multiple comparisons. Based on the statistical comparison, univariate logistic regression analysis was performed to identify possible risk factors for the development of chest wall rigidity, with the odds ratios (ORs) and corresponding 95% confidence intervals (CIs) for each variable. All data are expressed as the number of patients, means (SD), or numbers (percentages). A *P* value less than 0.05 was considered statistically significant.

## Results

**Fig. 1:**
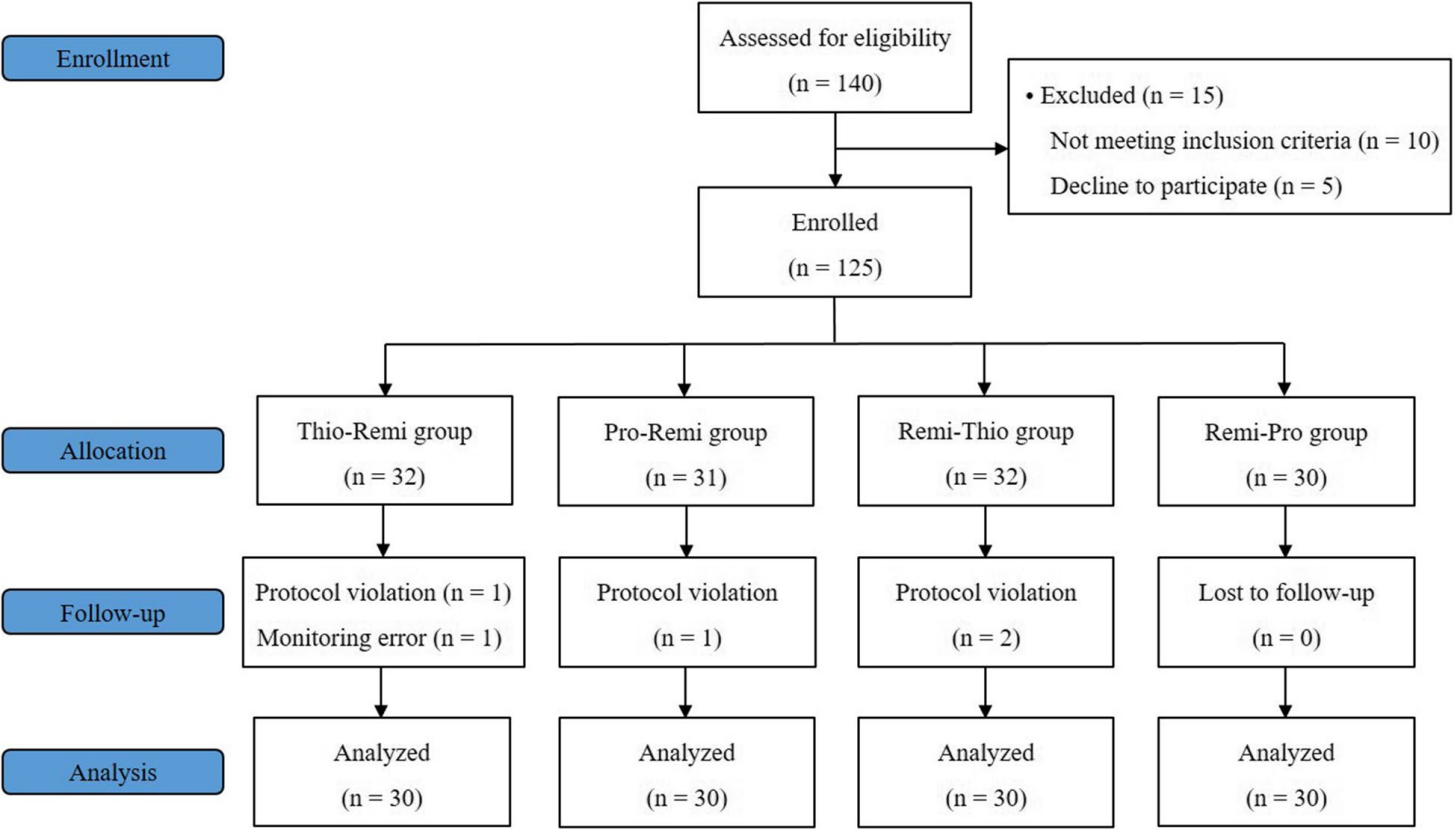
The CONSORT flow diagram

Among the 125 enrolled participants, 120 (30 in each group) were analyzed in the current study. The five participants who failed to complete the study included four who violated the study protocol and one with a SpO_2_ monitoring error from pulse oximetry. The CONSORT flow diagram is shown in Fig. 1.

Patient characteristics, including body composition measures, were compared among the Thio-Remi, Pro-Remi, Remi-Thio, and Remi-Pro groups (Table 1). No significant differences in patient age, gender, height, weight, or body mass index (BMI) were noted. Body composition measures, such as body fat mass, percent body fat, skeletal muscle mass, and total body water, were also not statistically different among the four groups. The hypnotic dose required for LOC and elapsed time to LOC were comparable among the groups (Table 2).

**Table 1:** Patient characteristics and body composition measures among the four groups

	Thio-Remi (*n* = 30)	Pro-Remi (*n* = 30)	Remi-Thio (*n* = 30)	Remi-Pro (*n* = 30)	*P* value
Age (years)	68.7 (4.7)	71.4 (5.9)	70.5 (5.2)	71.0 (6.1)	0.257
65–75 years	26	24	24	23	0.796
$\ge$ 75 years	4	6	6	7	
Female [n (%)]	14 (46.7%)	22 (73.3%)	18 (60.0%)	19 (63.3%)	0.205
Height (cm)	159.0 (9.3)	156.2 (6.0)	156.7 (9.5)	158.2 (7.7)	0.523
Weight (kg)	65.5 (9.9)	60.1 (9.1)	59.6 (9.8)	60.9 (11.7)	0.099
BMI (kg/m^2^)	25.9 (3.2)	24.7 (3.6)	24.2 (2.4)	24.2 (3.6)	0.134
Body fat mass (kg)	18.1 (6.3)	16.8 (6.7)	16.2 (4.9)	16.2 (7.1)	0.657
Percent body fat (%)	27.4 (9.3)	27.7 (7.9)	27.8 (8.6)	26.1 (9.0)	0.858
Skeletal muscle mass (kg)	26.6 (6.0)	23.3 (4.4)	23.4 (6.2)	24.3 (5.1)	0.098
Skeletal muscle index (kg/m^2^)	8.4 (1.2)	7.7 (1.2)	7.5 (1.5)	7.7 (1.2)	0.049^*^
Total body water (L)	35.6 (7.2)	31.7 (5.5)	31.8 (7.7)	32.9 (6.3)	0.110
Underlying diseases [n (%)]					
Hypertension	15 (50.0%)	11 (36.7%)	16 (53.3%)	13 (43.3%)	0.576
Diabetes mellitus	4 (13.3%)	4 (13.3%)	8 (26.7%)	4 (13.3%)	0.410

Values are presented as means (SD), numbers, or numbers (percentages)

*BMI* Body mass index

^*^There were no significant differences based on multiple comparisons with Tukey’s post-hoc test

**Table 2:** Hypnotic requirements and elapsed time to loss of consciousness

	Thio-Remi (*n* = 30)	Pro-Remi (*n* = 30)	Remi-Thio (*n* = 30)	Remi-Pro (*n* = 30)	*P* value
Administered dose of thiopental (mg)	244.4 (77.0)	N/A	222.0 (60.2)	N/A	0.215
Administered dose of propofol (mg)	N/A	99.3 (14.7)	N/A	92.4 (25.0)	0.201
Elapsed time to LOC (s)	106.6 (26.4)	101.7 (15.2)	91.5 (24.5)	94.6 (25.8)	0.059

Values are presented as means (SD)

*N/A* Not applicable, *LOC* Loss of consciousness

**Fig. 2:**
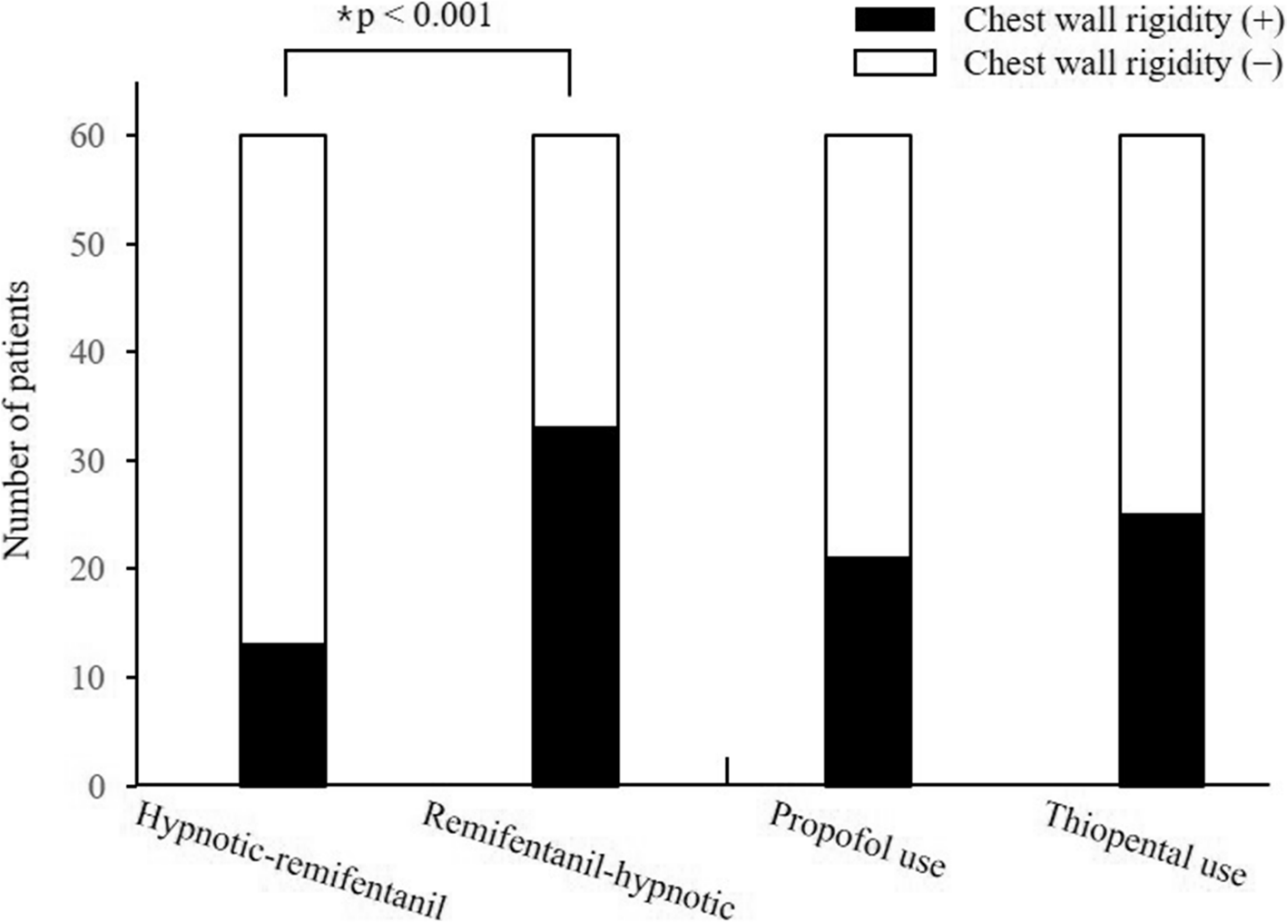
The number of patients experiencing chest wall rigidity according to the administration sequence of the hypnotics and remifentanil as well as the type of hypnotic. ^*^The numbers of patients who exhibited chest wall rigidity were 13/60 (21.7%) and 33/60 (55.0%) in the hypnotic-remifentanil and remifentanil-hypnotic groups, respectively (*P* < 0.001). There was no significant difference in the development of chest wall rigidity between propofol and thiopental sodium use

The incidence of chest wall rigidity and other adverse effects is presented in Table 3. Significant differences in the incidence of chest wall rigidity and desaturation were noted among the four groups (*P* = 0.001 and *P* = 0.034, respectively). The incidence of chest wall rigidity was significantly higher in the remifentanil-hypnotics groups than in the hypnotic-remifentanil groups (55.0% vs. 21.7%, *P* < 0.001 according to the Chi-square test) when compared according to the administration sequence of the hypnotics and remifentanil (Fig. 2). However, no significant difference in the development of chest wall rigidity was observed between propofol and thiopental sodium use. The univariate logistic regression analysis revealed that remifentanil-hypnotic administration was a significant predictor of the development of chest wall rigidity (crude OR 4.42, 95% CI 1.99; 9.81, *P* < 0.001) (Table 4).

**Table 3:** Incidence of remifentanil-induced chest wall rigidity and other adverse effects

	Thio-Remi (*n* = 30)	Pro-Remi (*n* = 30)	Remi-Thio (*n* = 30)	Remi-Pro (*n* = 30)	*P* value
Chest wall rigidity	9 (30%)	4 (13.3%)	16 (53.3%)	17 (56.7%)	0.001^*^
Desaturation	2 (6.7%)	2 (6.7%)	8 (26.7%)	8 (26.7%)	0.034^*^
Cough	2 (6.7%)	4 (13.3%)	1 (3.3%)	2 (6.7%)	0.516

Categorical variables are presented as numbers (%)

Desaturation is indicated by a SpO_2_ < 94%

^*^ *P* < 0.05 by chi-square test comparisons among the four groups

**Table 4:** Possible risk factors for remifentanil-induced chest wall rigidity based on logistic regression analysis

	Univariate analysis	
	Crude OR [95% CI]	*P* value
$\ge$ 75 years	1.85 [0.77–4.45]	0.170
Female sex	1.10 [0.52–2.35]	0.807
Remifentanil-hypnotic order	4.42 [1.99–9.81]	< 0.001^*^
Propofol usage	0.751 [0.36–1.58]	0.453
BMI (kg/m^2^)	1.06 [0.95–1.19]	0.315
Percent body fat (%)	1.00 [0.96–1.05]	0.848
Skeletal muscle index (kg/m^2^)	1.12 [0.84–1.50]	0.437

^*^Compared with the hypnotic-remifentanil sequence

**Fig. 3:**
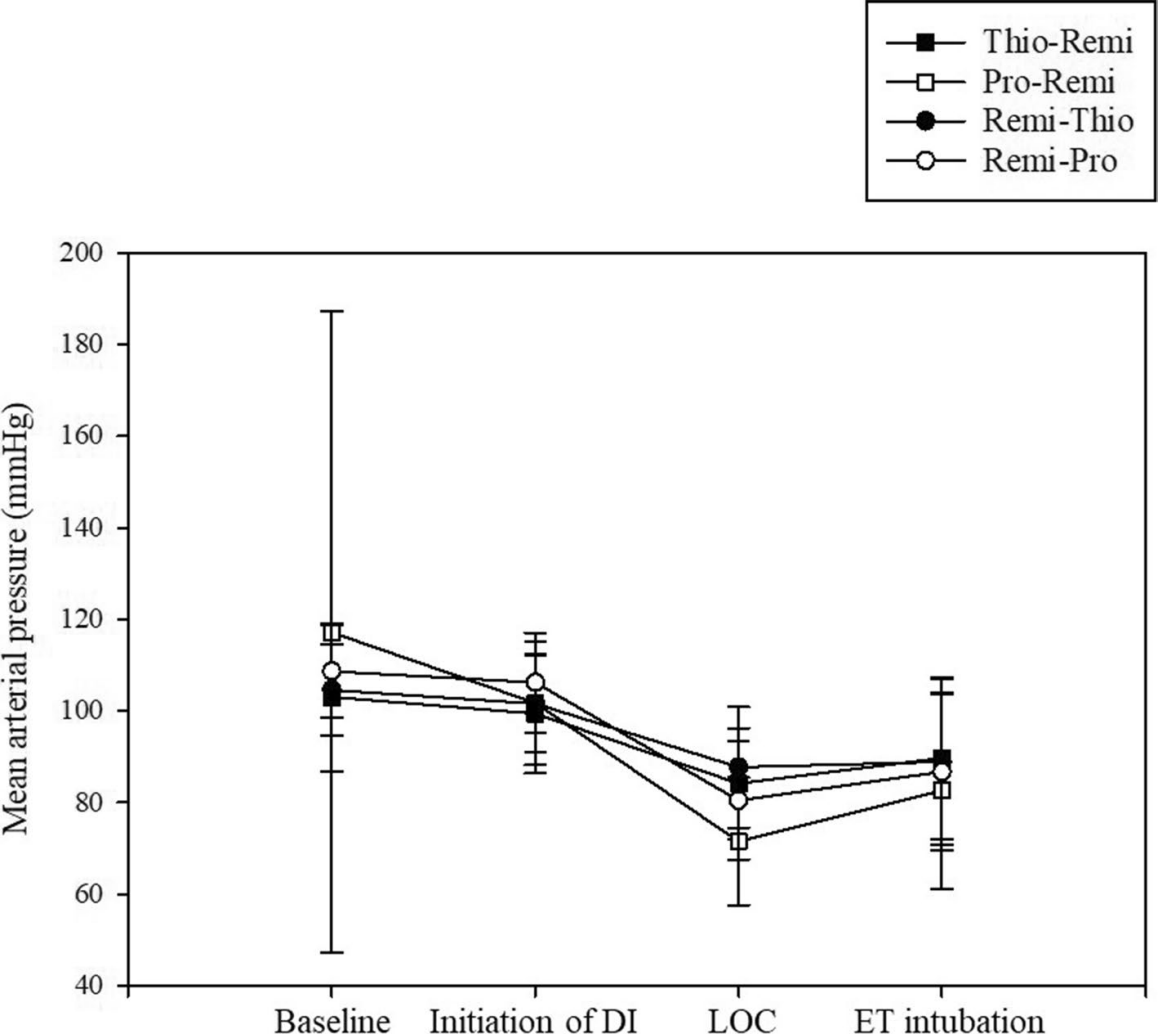
The changes in mean arterial pressures among the four groups. DI; drug infusion, LOC; loss of consciousness, ET; endotracheal

**Fig. 4:**
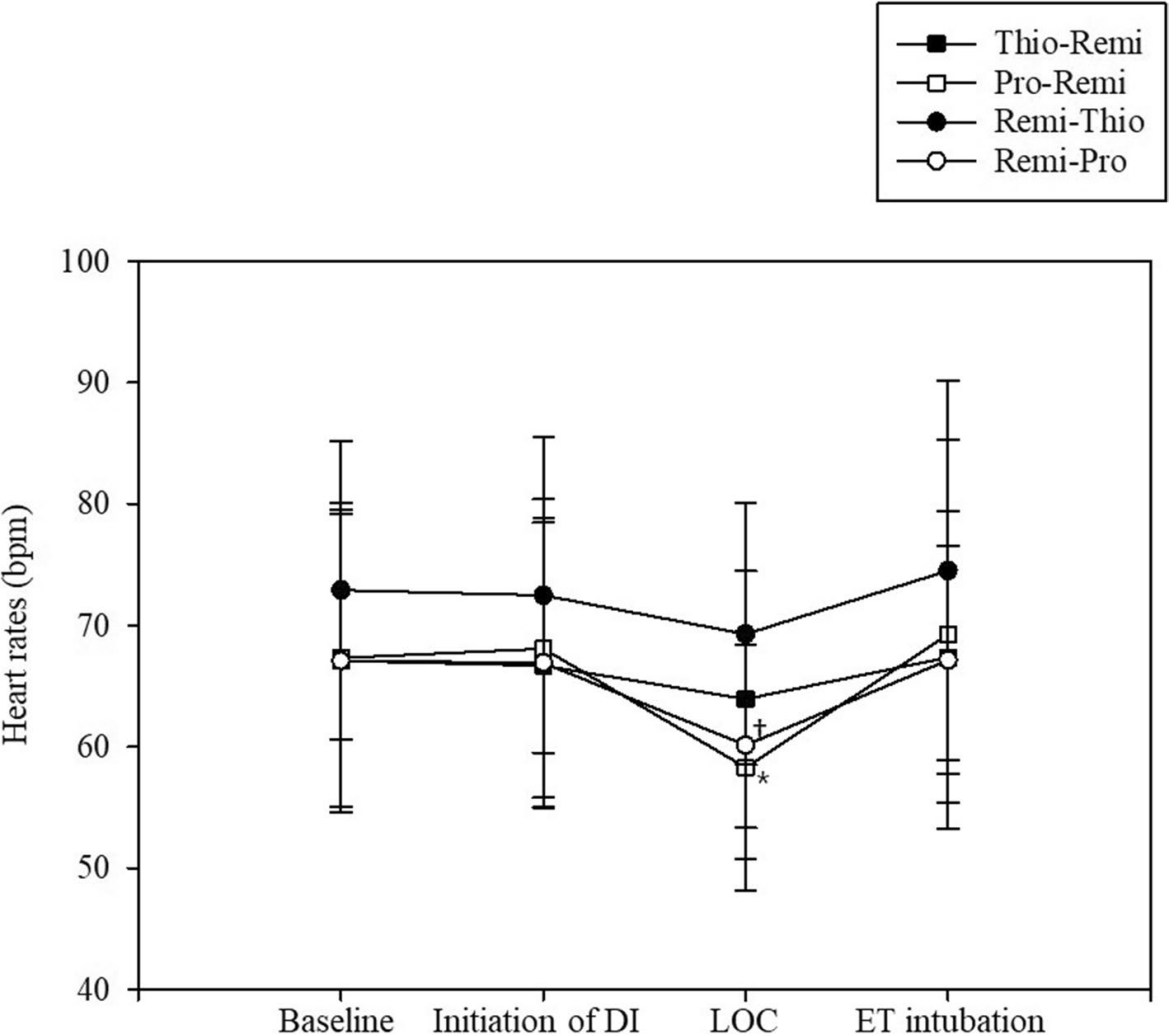
The changes in heart rates among the four groups. DI; drug infusion, LOC; loss of consciousness, ET; endotracheal. ^*^Compared with that at baseline, the heart rate was significantly lower at LOC (*P* < 0.001) in the Pro-Remi group. ^†^The heart rate was also significantly lower at LOC (*P* = 0.001) than at baseline in the Remi-Pro group

Changes in vital signs, such as mean arterial pressure and heart rate, are shown in Figs. 3 and 4. Although no differences in heart rate were noted among the groups, the heart rate was significantly lower at LOC than at baseline in both the Pro-Remi and Remi-Pro groups (*P* < 0.001 and *P* = 0.001, respectively).

## Discussions

The current study characterized remifentanil-induced chest wall rigidity in older patients in usual clinical practice. In this study, the overall incidence of remifentanil-induced chest wall rigidity were 38% (46/120) in older patients (≥ 65 years), and SpO_2_ desaturation < 94% developed in 16.7% (20/120). Careful anesthetic management and close monitoring are required in older patients because they are more vulnerable to perioperative morbidity. This study demonstrated that remifentanil administration at a relatively low and conventional dosage potentially leads to severe chest wall rigidity in older patients, although it is commonly accepted that opioid–induced muscle rigidity develops more frequently when larger doses are administered rapidly.

The result of a heathy volunteer study showed that muscle rigidity developed in 50% of subjects who were administered high-dose fentanyl (15 μg/kg) [15]. Another study reported the incidence of difficult ventilation and respiratory failure to be 46% and 9.1%, respectively, in patients treated with high-dose remifentanil (0.7 μg/kg/min) during anesthesia induction. Moreover, they suggested that a relatively lower dose of remifentanil (0.2 μg/kg/min) resulted in significantly reduced incidence of them [6]. In the current study, 3 ng/ml of remifentanil via TCI system, which is the conventional dosage for anesthesia induction, was selected for the investigation. This concentration approximately corresponds to a bolus dosing of 0.75 μg/kg, followed by 0.1–0.15 μg/kg/min, which is not a high dosage, when the pre-programmed Minto model and effect-site targeting mode are used [16]. Nevertheless, the incidence of remifentanil-induced chest wall rigidity was similar to that in other previous studies [6, 15]. The authors suggest that the development of remifentanil-induced chest wall rigidity may not occur in a dose-dependent manner.

Furthermore, the order of administration of hypnotics and opioids predominantly depends on the anesthesiologists’ preference, based on their anesthetic experiences. Propofol, the most commonly used hypnotic, is known to cause injection pain [17]. Therefore, the administration of remifentanil before the injection of propofol is a commonly performed method to reduce the incidence and severity of propofol injection-related pain during anesthesia induction [17–19]. Several studies have suggested that the TCI of remifentanil at a Ce of 3–6 ng/ml may be safely used for this purpose without serious respiratory complications, such as respiratory depression [18, 20]. However, it is remarkable that pretreatment with remifentanil prior to hypnotic administration was potentially associated with a higher probability of chest wall rigidity compared with the opposite sequence (crude OR 4.42, 95% CI 1.99; 9.81, *P* < 0.001) in the current study. Furthermore, this study aimed to clarify the effects of the body composition characteristics, such as BMI, percent body fat, or skeletal muscle index on it. No significant association between body composition and the development of remifentanil-induced chest wall rigidity was observed.

Although the precise pathophysiologic mechanism underlying opioid-induced chest wall rigidity has not yet been completely elucidated, centrally mediated noradrenergic and cholinergic actions are considered to be the most explainable underlying molecular mechanism [21–23]. An animal study indicated that the activation of μ-opioid receptors in the locus coeruleus appeared to increase noradrenergic outflow by activating α1-adrenergic receptor [21]. In addition, the inhibition of gamma-aminobutyric acid (GABA) interneuron signaling in the locus coeruleus potentially plays a partial role in the increased muscle rigidity [24–26]. Other experimental and clinical studies have suggested that pretreatment of hypnotics, including barbiturates and benzodiazepine, could mitigate the severity of opioid-induced chest wall rigidity by enhancing GABAergic activation [21, 27]. The current study demonstrated that both propofol and thiopental sodium have beneficial effects in preventing remifentanil-induced chest wall rigidity when administered as premedication (hypnotic-remifentanil administration).

The study has certain limitations. First, quantitative and objective measurements for evaluating chest wall rigidity are limited. Esophageal pressure, as a useful surrogate of pleural and intrathoracic pressure, can be used to estimate lung and chest wall mechanics. However, the measurements are complicated by several technical factors, such as the appropriate placement of the esophageal balloon catheter [28, 29], and are often unavailable because chest wall rigidity might develop shortly after opioid administration [23]. In the current study, the authors attempted to detect the development of opioid-induced chest wall rigidity based on bedside clinical signs and symptoms. There is scarce evidence on that issue, and that clinical evaluation might introduce a bias. An integrated qualitative and quantitative methodological approach may be necessary and can contribute to improving future research practices. Second, we did not investigate the possibility of remifentanil-induced chest wall rigidity and resultant hypoxemia affecting perioperative outcomes. Although the decreased SpO_2_ was simultaneously resolved following endotracheal intubation and neuromuscular relaxation in the current study, cerebral desaturation in older patients may be an important issue because of tis possible association with neurologic complications, such as postoperative delirium and cognitive dysfunction [30, 31]. The clinical significance of cerebral desaturation in perioperative neurologic outcomes should be investigated in future studies.

## Conclusions

In conclusion, older patients frequently experience remifentanil-induced chest wall rigidity during anesthesia induction. The authors suggest that pretreatment with hypnotics may reduce the possibility of chest wall rigidity during the induction of balanced anesthesia with remifentanil in older patients.

## Data Availability

The data analyzed during the current study are available from the corresponding author on reasonable request.

## Abbreviations

LOC: Loss of consciousness
TCI: Target-controlled infusion
Ce: Effect-site concentration
SpO_2_: Peripheral oxygen saturation
ANOVA: Analysis of variance
BMI: Body mass index
GABA: Gamma-aminobutyric acid
